# Platelet-to-Portal Vein Width Ratio and Platelet-to-Spleen Thickness Ratio Can Be Used to Predict Progressive Liver Fibrosis Among Patients With HBV Infection With HBeAg-Negativity and a Normal ALT Level

**DOI:** 10.3389/fmed.2022.837898

**Published:** 2022-06-21

**Authors:** Mudan Feng, Lan Lei, Jian Xu, Yuzhi Shi, Wenfeng Yang

**Affiliations:** ^1^Department of Infectious Disease, Affiliated Hospital of North Sichuan Medical College, Nanchong, China; ^2^Department of Hepatology and Translation Medicine, Fuling Center Hospital of Chongqing City, Chongqing, China; ^3^Department of Infectious Disease, The People's Hospital of Yubei District of Chongqing City, Chongqing, China

**Keywords:** fibrosis stage, hepatitis B virus, normal ALT, platelet-to-portal vein width ratio, platelet-to-spleen thickness ratio

## Abstract

**Background:**

Some people infected with the hepatitis B virus (HBV) with a normal level of alanine aminotransferase (ALT) are at risk of disease progression. We evaluated the value of platelet-to-portal vein width ratio (PPR) and platelet-to-spleen thickness ratio (PSR) to predict progressive liver fibrosis among patients with HBV infection with HBV e antigen (HBeAg)-negativity and a normal ALT level.

**Methods:**

HBV surface antigen (HBsAg)-positive and HBeAg-negative individuals with a normal ALT level were enrolled. The inflammation grade (G) and fibrosis stage(S) were analyzed according to pathological features. Then, two groups (<S2 *vs*. ≥S2) among people with a normal ALT level were divided based on the pathological diagnosis, and the clinical characteristics were summarized.

**Results:**

Seventy-three individuals among 142 patients with HBsAg-positivity and HBeAg-negativity had a normal ALT level. Also, 83.56% (61/73) individuals showed progressive liver fibrosis (≥S2). The ALT level and aspartate aminotransferase (AST) between the two groups differed (21.01 ± 7.40 vs. 25.37 ± 7.90 U/L, *p* = 0.08; 29.49 ± 13.56 vs. 30.16 ± 21.88 U/L, *p* = 0.92, respectively). Portal-vein width, serum levels of albumin and globulin, AST-to-Platelet Ratio Index (APRI), and Fibrosis 4 (FIB-4) score were not significantly different between the two groups (*p* > 0.05). The platelet count, PPR, and PSR were significantly different between the two groups [(145.92 ± 14.55) ×10^9^/L vs. (126.38 ± 23.85) ×10^9^/L, *p* = 0.008; 10.80 ± 1.30 vs. 9.01 ± 1.97, *p* = 0.004; 4.21 ± 0.65 vs. 3.33 ± 0.89, *p* = 0.02, respectively]. The PPR and PSR decreased gradually upon fibrosis aggravation (*p* < 0.05). Based on the cut off value of the PPR (9.07) and PSR (3.54), their sensitivity and specificity was 0.917 and 0.525, and 0.833 and 0.541, respectively.

**Conclusion:**

The PPR and PSR can be employed to assess earlier fibrosis progression among patients with HBV infection with HBeAg-negativity and a normal ALT level.

## Introduction

The natural history of hepatitis B virus (HBV) infection can be divided into four stages: immune tolerance, immune clearance, inactive carrier status, and reactivity (1). “Immune tolerance” refers to the non-responsive state after the immune system of the body meets a specific antigen (2). Previously, it was believed that most patients with chronic HBV infection were in the “immune tolerance period” (ITP) (3). Patients in the ITP have slight inflammatory necrosis and/or liver fibrosis, and a poor response to antiviral therapy. Simultaneously, spontaneous HBV e antigen (HBeAg) serological conversion and sustained remission are possible during the ITP. Otherwise, long-term antiviral therapy in the ITP can lead to drug resistance and other adverse events, and even increase the economic burden of patients. Therefore, most patients with immune tolerance of chronic HBV infection do not need antiviral therapy (4, 5).

There is a lack of sensitive and specific markers to measure the immune tolerance of patients after HBV infection. However, in the definition of the ITP of HBV infection by various hepatology societies, a continuously normal level of alanine aminotransferase (ALT) is regarded as a basic characteristic (6–8). However, there is also evidence that significant fibrosis occurs in a large proportion of HBV-infected patients with a normal ALT level who are considered ITP (9). Hepatocellular carcinoma has been found in some patients in the ITP (10, 11). Liver histopathology of HBV-infected people with a normal ALT level indicates that about one-third to one-half of cases are not in the ITP (12–14). Therefore, clinicians must judge if patients of chronic HBV infection with a normal ALT level are in the ITP.

Liver biopsy is the “gold standard” for determining if the liver has inflammation and fibrosis. However, ascertaining if a patient has liver inflammation by liver biopsy is impractical because it is invasive. Accurate assessment of the degree of liver fibrosis in patients with a normal ALT level in a non-invasive manner is crucial to ascertain if patients are immune-tolerant.

We investigated the basic clinical and laboratory characteristics of progressive liver fibrosis among patients with HBV infection who were HBeAg-negative and had a normal ALT level.

## Patients and Methods

### Ethical Approval of the Study Protocol

The study protocol was approved by the Clinical Research Ethics Committee of Fuling Center Hospital of Chongqing City (Chongqing, China). Written informed consent was obtained from patients (or their legal surrogates) before data collection.

### Inclusion Criteria

The inclusion criteria were: (i) serum hepatitis B surface antigen (HBsAg)-positivity for ≥6 months; (ii) serum HBeAg-negativity; (iii) never treated with antiviral agents.

### Exclusion Criteria

The exclusion criteria were: (i) infection with hepatitis A, C, D, E or other viruses; (ii) history of alcohol and/or drug intake that caused liver damage; (iii) non-alcoholic fatty liver disease; (iv) magligant liver tumor and definite cirrhosis; (v) history of hereditary metabolic liver disease or autoimmune liver disease; (vi) history of hypertension, diabetes mellitus, coronary heart disease, or metabolic syndrome; (vii) history of traumatic fractures.

### Study Design

**Figure 1:**
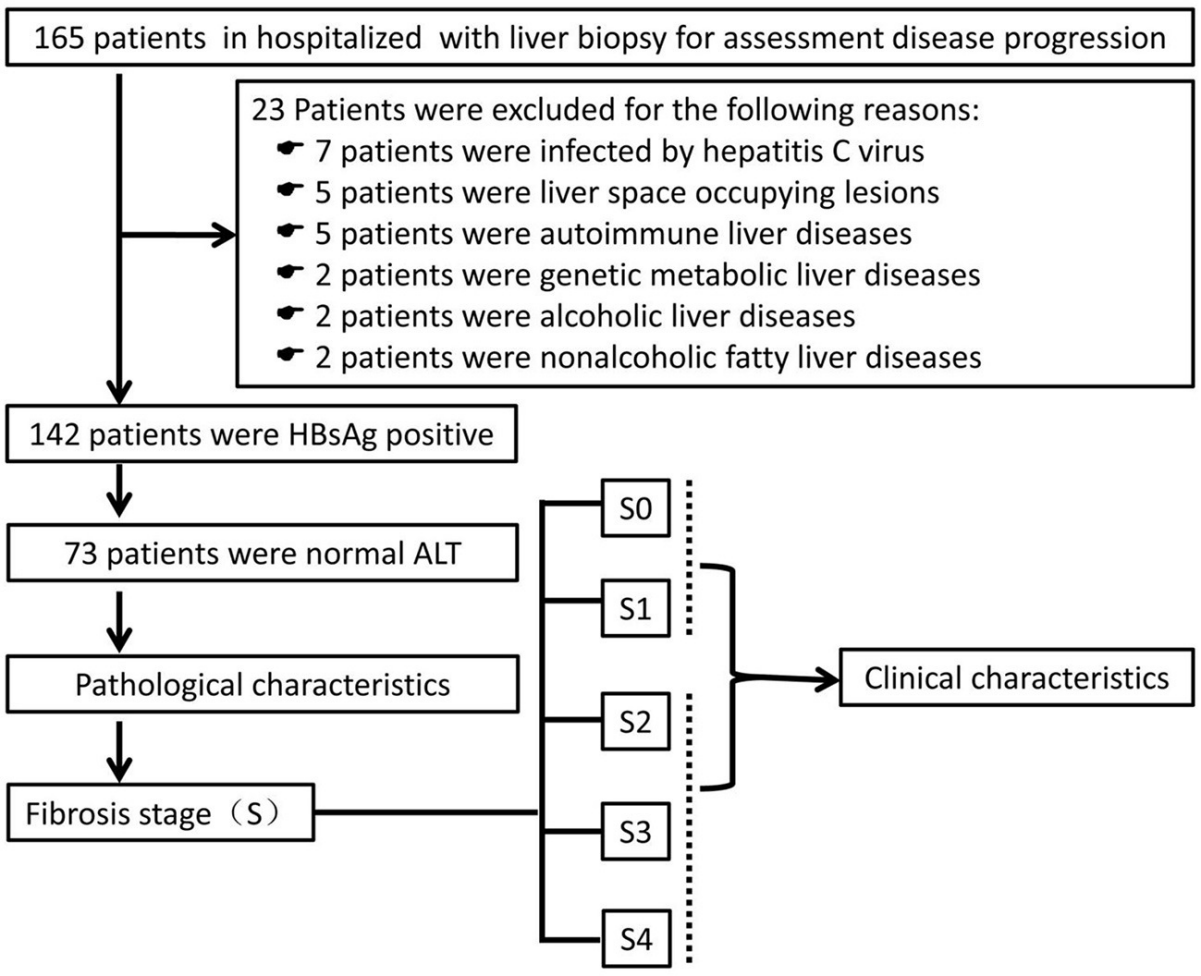
Screening, enrollment and grouping of the study. ALT, alanine aminotransferase; HBsAg, hepatitis B surface antigen.

A total of 165 patients who underwent liver biopsy for assessment of disease progression from July 2019 to June 2021 at Fuling Center Hospital of Chongqing City were evaluated, retrospectively (Figure 1). Twenty-three patients were excluded. Hence, 142 patients with HBsAg-positivity and HBeAg-negativity were enrolled. Of these, 73 patients had a normal ALT level (defined as ALT <40U/L). All patients underwent ultrasound-guided percutaneous liver biopsy followed by calculation of the METAVIR score to assess the inflammation grade (G) and fibrosis stage (S). Then, patients were divided into two groups: progressive liver fibrosis (S ≥ 2) and non-progressive fibrosis (S <2).

### Data

The clinical and laboratory data of all patients were collected upon hospital admission. These data comprised: age; platelet count; serum levels of ALT, aspartate aminotransferase (AST), albumin, globulin, and albumin/globulin (A/G) ratio; portal vein width (PVW); spleen thickness.

Serum levels of HBeAg or HBsAg were quantified using a standardized electro-chemiluminescent immunoassay (Architect™ HBeAg; Abbott, Chicago, IL, USA). HBV DNA was measured using COBAS TaqMan v2.0 (limit of detection = 20 IU/mL; Roche, Basel, Switzerland). Laboratory tests were done upon admission to Fuling Center Hospital of Chongqing City.

### Liver Histology

Ultrasound-guided percutaneous liver puncture was carried out. Then, hematoxylin and eosin-stained liver biopsies were tested by two specialist pathologists in a blinded manner. G ≥ 2 was considered “moderate/severe necroinflammation”, and S ≥ 2 was regarded as “liver fibrosis progression state” according to the METAVIR scoring system (15–17).

### Statistical Analyses

SPSS 22.0 (IBM, Armonk, NY, USA) was used for statistical analyses. Continuous variables (presented as frequencies and percentages or the mean ± SD) were compared using the Student's *t*-test or non-parametric Mann–Whitney *U*-test. Categorical data were analyzed with the chi-squared test and Fisher's exact test. Receiver-operating characteristic (ROC) curves were generated and the area under the ROC curve (AUC) was calculated and compared. Binary logistic regression was employed to consider the cutoff value. Optimal cutoff values were selected to maximize specificity and sensitivity. *P* < 0.05 was considered significant.

## Results

### Patient Characteristics

Twenty three patients of the 165 patients were excluded: infection with the hepatitis C virus, 7 patients; space-occupying lesion in the liver, 5 patients; autoimmune liver disease, 5 patients; genetic metabolic liver disease, 2 patients; alcoholic liver disease, 2 patients; non-alcoholic fatty liver disease, 2 patients. Thus, 142 patients were enrolled in this study, and 73 individuals had a normal ALT level (Figure 1).

### Inflammation Grade and Fibrosis Stage of 142 Patients

Of 142 patients, the number with an inflammation grade of 0, 1, 2, 3, and 4 was 0, 16, 100, 23, and 3, respectively (Table 1). For patients with inflammation grade 0, 1, 2, 3, and 4, the percentage with a fibrosis stage ≥2 was 0, 18.75, 93, 100, and 100%, respectively. Overall, 83.80% (119/142) of individuals had progressive liver fibrosis (S ≥2).

**Table 1:** Inflammation grade and fibrosis stage of 142 patients with HBsAg-positivity and HBeAg-negativity.

**Inflammation grade(G)**		**Fibrosis stage (S)**		**≥G2S2**
**G**	***N*** **(%)**	**S**	***N*** **(%)**	***N*** **(%)**
G0	0 (0)	S0	0 (0)	119 (83.80%)
		S1	0 (0)	
		S2	0 (0)	
		S3	0 (0)	
		S4	0 (0)	
G1	16 (11.27)	S0	9 (56.25)	
		S1	4 (25.00)	
		S2	2 (12.50)	
		S3	1 (6.25)	
		S4	0 (0)	
G2	100 (70.42)	S0	0 (0)	
		S1	7 (7.00)	
		S2	55 (55.00)	
		S3	25 (25.00)	
		S4	13 (13.00)	
G3	23 (16.20)	S0	0 (0)	
		S1	0 (0)	
		S2	0 (0)	
		S3	15 (65.22)	
		S4	8 (34.78)	
G4	3 (2.11)	S0	0 (0)	
		S1	0 (0)	
		S2	0 (0)	
		S3	1 (33.33)	
		S4	2 (66.67)	

*HBsAg, hepatitis B surface antigen*.

### Inflammation Grade and Fibrosis Stage of 73 Patients

For the 73 patients with a normal ALT level, the number with an inflammation grade of 0, 1, 2, 3, and 4 was 0, 10, 56, 7, and 0, respectively. For patients with inflammation grade 0, 1, 2, 3, and 4, the percentage with a fibrosis stage ≥2 was 0, 10.00, 94.64, 100, and 0%, respectively. Overall, 83.56% (61/73) patients had **progressive** liver fibrosis (S ≥ 2) (Table 2).

**Table 2:** Inflammation grade and fibrosis stageamong73 HBV-infected patients with HBeAg-negativity and a normal ALT level.

**Inflammation grade(G)**		**Fibrosis stage (S)**		**≥G2S2**
**G**	***N*** **(%)**	**S**	***N*** **(%)**	***N*** **(%)**
G0	0 (0)	S0	0 (0)	61 (83.56%)
		S1	0 (0)	
		S2	0 (0)	
		S3	0 (0)	
		S4	0 (0)	
G1	10 (13.70)	S0	7 (70.00)	
		S1	2 (20.00)	
		S2	0 (0)	
		S3	1 (10.00)	
		S4	0 (0)	
G2	56 (56.71)	S0	0 (0)	
		S1	3 (5.36)	
		S2	32 (57.14)	
		S3	16 (28.57)	
		S4	5 (8.93)	
G3	7 (9.59)	S0	0 (0)	
		S1	0 (0)	
		S2	0 (0)	
		S3	6 (85.71)	
		S4	1 (14.29)	
G4	0 (0)	S0	0 (0)	
		S1	0 (0)	
		S2	0 (0)	
		S3	0 (0)	
		S4	0 (0)	

*ALT, alanine aminotransferase; HBeAg, hepatitis B e antigen; HBV, hepatitis B virus*.

### Characteristics of Progressive Liver Fibrosis Among People With HBV Infection Who Were HBeAg-Negative With a Normal ALT Level

Levels of ALT and AST were not significantly different between the group with fibrosis stage <2 and those with fibrosis stage ≥2 (21.01 ± 7.40 vs. 25.37 ± 7.90 U/L, *p* = 0.08; 29.49 ± 13.56 vs. 30.16 ± 21.88 U/L, *p* = 0.92, respectively). PVW, albumin level, globulin level, A/G ratio, APRI, and FIB-4 score were not significantly different between the group with fibrosis stage <2 and the group with fibrosis stage ≥2 (*p* > 0.05). The platelet count, platelet-to-portal vein width ratio (PPR), and platelet-to-splenomegaly ratio (PSR) were significantly different between the group with fibrosis stage <2 and the group with fibrosis stage ≥2 [(145.92 ± 14.55) ×10^9^/L vs. (126.38 ± 23.85) ×10^9^/L, *p* = 0.008; 10.80 ± 1.30 vs. 9.01 ± 1.97, *p* = 0.004; 4.21 ± 0.65 vs. 3.33 ± 0.89, *p* = 0.02, respectively] (Table 3).

**Table 3:** Characteristics of liver-fibrosis progression among HBV-infected patients with HBeAg-negativity and a normal ALT level.

**Factors**	**Unit**	**Stage <2** **(*n* = 12)**	**Stage ≥**2 **(*n* = 61)**	***t***	***p***
Age	years	50.08 ± 4.80	47.72 ± 7.96	0.99	0.325
ALT	U/L	21.01 ± 7.40	25.37 ± 7.90	−1.765	0.08
AST	U/L	29.49 ± 13.56	30.16 ± 21.88	−0.101	0.92
PLT	×10^9^/L	145.92 ± 14.55	126.38 ± 23.85	2.73	0.008
PVW	mm	13.59 ± 1.38	14.21 ± 1.91	−1.081	0.283
Spleen thickness	mm	35.22 ± 5.56	39.39 ± 8.80	−1.576	0.12
ALB	g/L	44.77 ± 2.41	44.15 ± 5.65	0.617	0.541
GLO	g/L	30.08 ± 5.53	30.83 ± 6.18	−0.387	0.7
A/G	–	1.52 ± 0.33	1.47 ± 0.31	0.452	0.652
APRI	–	0.52 ± 0.25	0.63 ± 0.49	−0.781	0.434
FIB-4 score	–	2.23 ± 60.84	2.38 ± 1.47	−0.322	0.748
PPR	–	10.80 ± 1.30	9.01 ± 1.97	3.012	0.004
PSR	–	4.21 ± 0.65	3.33 ± 0.89	3.241	0.02

*ALB, serum albumin; ALT, alanine aminotransferase; APRI, AsparteAminotransferase-to-Platelet Ratio Index; AST, aspartate aminotransferase; FIB-4, Fibrosis 4; GLO, serum globulin; HBeAg, hepatitis B e antigen; HBV, hepatitis B virus; PLT, platelet count; PPR, platelet-to-portal vein width ratio; PSR, platelet-to-spleen thickness ratio; PVW, portal-vein width*.

### PPR and PSR in Progressive Liver Fibrosis Among People With HBV Infection, HBeAg-Negativity, and a Normal ALT Level

The PPR and PSR decreased gradually with aggravation of liver fibrosis (*p* < 0.05) (Table 4). Binary logistic regression analysis revealed the PPR and PSR to be independent risk factors for advanced fibrosis among people with HBV infection and HBeAg-negativity and a normal ALT level. Analysis of ROC curves was applied to evaluate the performance of the PPR and PSR for prediction of progressive liver fibrosis among people with HBV infection with HBeAg-negativity and a normal ALT level.

**Table 4:** PPR and PSR in advanced liver fibrosis among HBV-infected patients with HBeAg-negativity and a normal ALT level.

**S**	***N***	**PPR**				**PSR**			
		**Mean ±SD**	**95%CI**	***F***	***p***		**Mean ±SD**	**95%CI**	***F***	***p***
S0	7	10.71 ± 1.02	(9.76, 11.66)	4.11	0.005		4.18 ± 0.51	(3.70, 4.65)	3.05	0.023
S1	5	10.94 ± 1.74	(8.78, 13.10)				4.27 ± 0.87	(3.19, 5.35)		
S2	32	9.53 ± 1.71	(8.91, 10.14)				3.39 ± 0.71	(3.14, 3.65)		
S3	23	8.66 ± 2.13	(7.74, 9.59)				3.38 ± 1.11	(2.90, 3.86)		
S4	6	7.61 ± 2.00	(5.51, 9.71)				2.89 ± 0.79	(2.06, 3.72)		

*ALT, alanine aminotransferase; HBeAg, hepatitis B e antigen; HBV, hepatitis B virus; N, number; PPR, platelet-to-portal vein width ratio; PSR, platelet-to-spleen thickness ratio; S, Fibrosis stage; 95%CI,95% confidence interval*.

**Figure 2:**
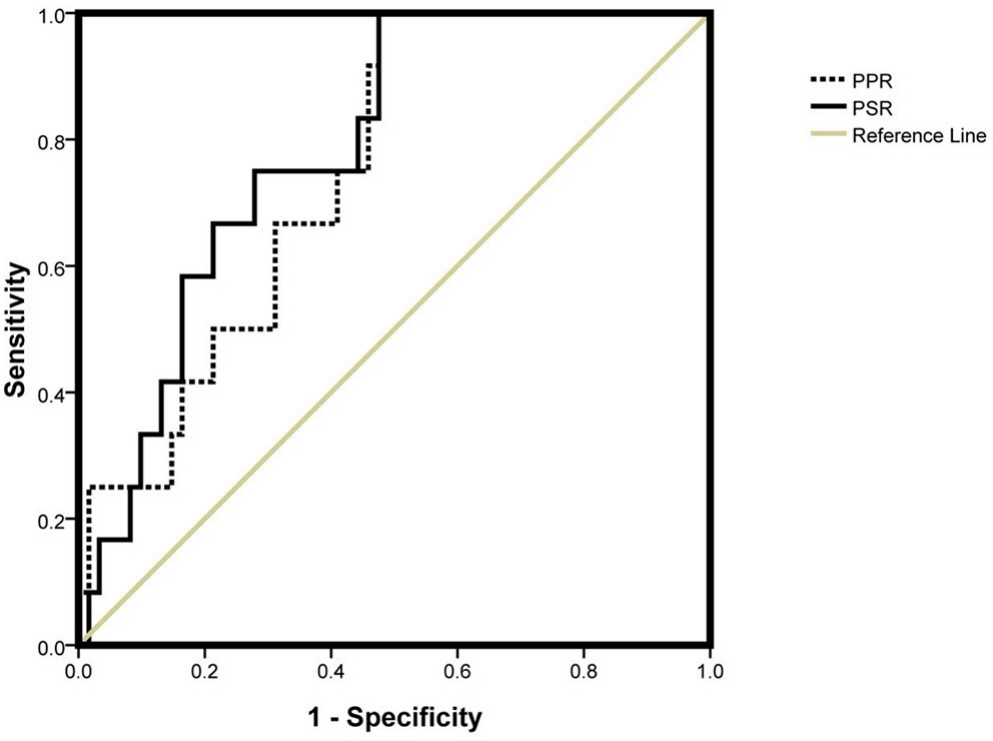
AUC of the PPR and PSR. PPR, platelet-to-portal vein width ratio; PSR, platelet-to-spleen thickness ratio.

The AUC of the PPR and PSR was 0.751 and 0.786, respectively (Figure 2). The optimal cut off value of the PPR and PSR was 9.07 and 3.54, respectively. The corresponding sensitivity and specificity of the PPR were 0.917 and 0.525, and those of the PSR were 0.833 and 0.541, respectively (Table 5).

**Table 5:** Efficacy of the PPR and PSR for evaluating hepatic-fibrosis progression among HBV-infected patients with HBeAg-negativity and a normal ALT level.

	**Cutoff**	**AUC**	**95%CI**	**Sensitivity**	**Specificity**	***p***
PPR	9.07	0.751	(0.627, 0.876)	0.917	0.525	0.006
PSR	3.54	0.786	(0.669, 0.902)	0.833	0.541	0.002

*ALT, alanine aminotransferase; AUC, area under the curve; HBeAg, hepatitis B e antigen; HBV, hepatitis B virus; PPR, platelet-to-portal vein width ratio; PSR, platelet-to-spleen thickness ratio; 95%CI, 95% confidence interval*.

## Discussion

Approximately 257 million people are infected by the HBV worldwide (18). Recent studies have shown that ~19% of people infected with the HBV require antiviral treatment (19). For chronic HBV infection, antiviral therapy can prevent the progression of fibrosis, cirrhosis, and cancer of the liver, and reduce the mortality associated with liver disease. Hence, antiviral therapy is crucial (20–22).

The serum level of ALT reflects the host immunity to viral challenge, and is one of the most sensitive biomarkers for liver inflammation. Therefore, an increase in the serum level of ALT is a good indication of hepatic necroinflammation, and is one of the crucial indexed to evaluate disease status for CHB patients (23), and is one of the parameter for determining initiation of antiviral therapy (8, 24, 25). However, in real-world clinical practice, the ALT level is not completely consistent with the degree of liver inflammation and progressive fibrosis. Inflammation and fibrosis as judged by histology has been documented in people with a HBV infection and a normal level of ALT (6, 26, 27).

We found that 83.80% (119/142) of individuals with an HBV infection had progressive fibrosis. Simultaneously, 83.56% (61/73) of patients with a normal ALT level had progressive fibrosis (fibrosis stage ≥2). ALT is a marker of liver dysfunction. In China, about 62.4% of HBV-infected patients have a normal ALT level, and these patients are often mistaken for having immune tolerance. Changes in the ALT level are not always consistent with the degree of inflammation of liver tissue. Hence, errors may arise in judging if patients need antiviral therapy based on the ALT level. Therefore, a normal ALT level cannot be used to ascertain if patients need treatment.

Liver biopsy is the gold standard to assess fibrosis/cirrhosis in HBV patients objectively (7). Antiviral therapy should be applied to HBV patients with a Knodell Histology Activity Index ≥4 or moderate/severe necroinflammation and/or fibrosis with a normal ALT level (20, 21). We observed that, in HBV-infected patients with a normal ALT level, the progression of inflammation and fibrosis could not be determined by hepatic biochemical indices (ALT, AST, albumin, globulin, A/G ratio), imaging (PVW, spleen thickness), or other characteristics (e.g., age) alone. These data suggest that we should reconsider the guiding role of the ALT level for starting antiviral therapy in HBV patients. We also under took evaluation by non-invasive methods, such as the APRI and FIB-4 Score. These are recommended by the World Health Organization as non-invasive evaluations of inflammation and fibrosis of the liver. However, these two indices are not applicable to HBV-infected patients with a normal ALT level.

We noted a significant difference in the platelet count between patients with fibrosis stage <2 and patients with fibrosis stage ≥2. Hence, fibrosis progression in HBV-infected patients with a normal ALT level was inversely proportional to the number of platelets. Thus, although the ALT was normal in HBV-infected patients, inflammation and progressive fibrosis may be present in the liver tissue. Patients with progressive liver fibrosis require antiviral therapy regardless of ALT status (8, 20, 28). Early antiviral therapy in these patients could also achieve a greater immune response (29). Our results suggest that HBV-infected patients with a normal ALT level may also have significant hepatic pathological changes and a high risk of liver cancer or death.

Thrombocytopenia has been observed in 76–85% of patients with progressive liver disease. Thrombocytopenia is secondary to hypersplenism, possible immune-mediated mechanisms, direct viral suppression of platelet production, and reduced production of thrombopoietin (30, 31). Thrombocytopenia in individuals with chronic liver disease is associated with portal venous pressure and hypersplenism. The platelet number is influenced by many factors, whereas the portal-vein width and spleen thickness are mainly associated with portal hypertension during liver fibrosis/cirrhosis. We combined the platelet count with the portal-vein width and spleen thickness into the PPR and PSR. The PPR and PSR could be used to predict progressive fibrosis among HBV-infected patients with HBeAg-negativity and a normal ALT level. We calculated the sensitivity of the PPR and PSR to be 0.917 and 0.833, respectively, where as the specificity of the PPR and PSR was only 0.525 and 0.541, respectively. Such low specificity may be related to the many factors influencing thrombocytopenia, as well as our small study cohort.

Liver biopsy is invasive, so timely and accurate assessment of the need for early liver biopsy is extremely important to detect progressive liver fibrosis. We demonstrated that patients with progressive fibrosis had a lower PPR and PSR. Further subgroup analysis showed that the PPR and PSR tended to decline gradually with fibrosis progression. Therefore, the PPR and PSR could reflect the stages of progressive fibrosis among HBV-infected patients with HBeAg-negativity and a normal ALT level. Using the PPR and PSR might provide better predictive accuracy than using the ALT level alone, especially for determining who needs a liver biopsy. In other words, the clinical guiding significance of PPR and PSR lies in that for HBV infected patients with a normal ALT and HBeAg negative, the PPR and PSR of patients should be closely observed. If the PPR of these patients is lower than 9.07 and PSR is lower than 3.54, liver biopsy should be mobilized as far as possible to obtain evidence of liver fibrosis progression in HBV infected patients. In contrast, if the appeal criteria are not met, follow-up may necessary and liver biopsy may not be required for the time being. It has certain guiding significance for saving medical resources and avoiding expanding indications of liver biopsy.

Our study had three main limitations. First, the study cohort was small, which hampered the robustness of statistical analyses. The small sample size was because liver biopsy is invasive and many patients refuse it. Second, our cohort may be not representative of all HBV-infected patients. Third, we excluded HBeAg-positive patients. Greater efforts should be made to explore more non-invasive methods to enable accurate judgment of disease progression for patients with a normal ALT level.

## Conclusions

Our study revealed the value of using the PPR and PSR for predicting progressive fibrosis among patients with HBV infection with HBeAg-negativity and a normal ALT level. The PPR and PSR could help to screen patients who need a liver biopsy for the time being.

## Data Availability Statement

The original contributions presented in the study are included in the article/supplementary material, further inquiries can be directed to the corresponding author.

## Ethics Statement

The study protocol was approved by the Clinical Research Ethics Committee of Fuling Center Hospital of Chongqing City (Chongqing, China). Written informed consent to participate in this study was obtained from patients (or their legal surrogates) before data collection.

## Author Contributions

LL and YS collected the data. MF carried out data analyses. JX contributed to the conception and design of the study. WY drafted and revised the manuscript. All authors approved the final version of the manuscript.

## Conflict of Interest

The authors declare that the research was conducted in the absence of any commercial or financial relationships that could be construed as a potential conflict of interest.

## Publisher's Note

All claims expressed in this article are solely those of the authors and do not necessarily represent those of their affiliated organizations, or those of the publisher, the editors and the reviewers. Any product that may be evaluated in this article, or claim that may be made by its manufacturer, is not guaranteed or endorsed by the publisher.
